# Epilepsy in *Dcx* Knockout Mice Associated with Discrete Lamination Defects and Enhanced Excitability in the Hippocampus

**DOI:** 10.1371/journal.pone.0002473

**Published:** 2008-06-25

**Authors:** Marika Nosten-Bertrand, Caroline Kappeler, Céline Dinocourt, Cécile Denis, Johanne Germain, Françoise Phan Dinh Tuy, Soraya Verstraeten, Chantal Alvarez, Christine Métin, Jamel Chelly, Bruno Giros, Richard Miles, Antoine Depaulis, Fiona Francis

**Affiliations:** 1 INSERM, U513, Université Pierre et Marie Curie, Paris, France; 2 UMPC Université Paris 06, Neurobiologie et Psychiatrie, Paris, France; 3 Institut Cochin, Université Paris Descartes, CNRS (UMR 8104), Paris, France; 4 INSERM, U567, Paris, France; 5 INSERM, U739, UPMC, CHU Pitié Salpêtrière, Paris, France; 6 UPMC, Paris, France; 7 INSERM, U839, Institut du Fer à Moulin, Paris, France; 8 Douglas Hospital Research Center, Department of Psychiatry, McGill University, Montreal, Quebec, Canada; 9 Grenoble Institute of Neurosciences, Inserm U836-UJF-CEA-CHU, Université Joseph Fourier, Grenoble, France; Chiba University Center for Forensic Mental Health, Japan

## Abstract

Patients with *Doublecortin* (*DCX)* mutations have severe cortical malformations associated with mental retardation and epilepsy. *Dcx* knockout (KO) mice show no major isocortical abnormalities, but have discrete hippocampal defects. We questioned the functional consequences of these defects and report here that *Dcx* KO mice are hyperactive and exhibit spontaneous convulsive seizures. Changes in neuropeptide Y and calbindin expression, consistent with seizure occurrence, were detected in a large proportion of KO animals, and convulsants, including kainate and pentylenetetrazole, also induced seizures more readily in KO mice. We show that the dysplastic CA3 region in KO hippocampal slices generates sharp wave-like activities and possesses a lower threshold for epileptiform events. Video-EEG monitoring also demonstrated that spontaneous seizures were initiated in the hippocampus. Similarly, seizures in human patients mutated for *DCX* can show a primary involvement of the temporal lobe. In conclusion, seizures in *Dcx* KO mice are likely to be due to abnormal synaptic transmission involving heterotopic cells in the hippocampus and these mice may therefore provide a useful model to further study how lamination defects underlie the genesis of epileptiform activities.

## Introduction

Doublecortin (DCX) is a microtubule-associated protein [1], [2] which is critical for human cortical development. Type I lissencephaly, due to *DCX* [3], [4] or *LIS*1 [5] mutations, is associated with severe mental retardation and refractory epilepsy [6]. This form of cortical dysplasia is due most probably to neuronal migration abnormalities during development [7]. In type 1 lissencephaly patients the neocortex has a smooth surface and generally consists of four disorganized layers of neurons instead of the six highly organized layers present in a normal brain [7]. Furthermore the hippocampus is disorganized [8]. The exact causes of epilepsy and mental retardation in these cases remain to be determined. However, epileptic seizures are likely to result from abnormal connectivity associated with the aberrant positioning of cortical neurons [9], [10].

*Dcx* KO mice have no obvious lamination defects in the isocortex [11], [12]. Indeed, radial migration of isocortical pyramidal cells appears to occur correctly leading to normally organized cortical layers. However, in the hippocampus of *Dcx* KO mice, the CA3 region of the pyramidal layer is disorganized and divided into at least two distinct layers [8], [11]. This is due to a slowed or arrested migration of a proportion of hippocampal pyramidal neurons at late embryonic stages [8]. Cortical interneuron migration abnormalities have also been identified with *Dcx* inactivation [12], [13]. Thus tangentially migrating neurons derived from the medial ganglionic eminence of *Dcx* KO or RNAi-treated mice show abnormal morphologies during migration in explant and slice cultures. Interneuron numbers were also found to be reduced in the isocortex of P0 animals [13]. With limited pyramidal cell and interneuron defects, *Dcx* KO mice may be a useful model to investigate the pathological processes of cognitive impairment, seizure susceptibility and epilepsy, independent of major isocortical lamination defects.

In the present study we show that *Dcx* KO animals are hyperactive and prone to epileptic seizures. This latter phenotype may be associated with changes in the expression of calbindin (CB), neuropeptide Y (NPY) and calretinin in the hippocampus. In addition, KO mice show an increased susceptibility to convulsants (pentylenetetrazole, PTZ and kainic acid, KA). Video-EEG monitoring demonstrated that spontaneous electrographical and behavioural seizures occur in the absence of convulsants. These appear to be initiated in the hippocampus and to rapidly diffuse to the cortex. Electrophysiological recordings *in vitro* revealed a lower threshold for epileptiform discharges and a higher frequency of spontaneous, sharp wave-like events in hippocampal slices from *Dcx* KO animals. Thus, *Dcx* KO mice, without severe isocortical disorganization, are susceptible to epilepsy most probably due to abnormal cell positioning, connectivity and synaptic transmission in the hippocampus.

## Results

### Morphological Abnormalities in the Hippocampus of *Dcx* KO Mice

**Figure 1:**
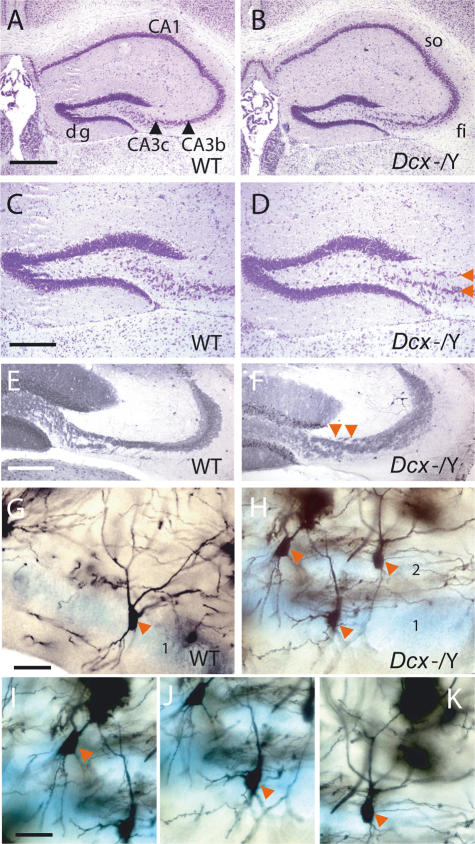
Morphological Abnormalities in the *Dcx* KO Hippocampus. (A–D) Cresyl violet staining showed that dentate gyri were indistinguishable between the genotypes, however, abnormally organized CA3 pyramidal cells in the KO hippocampus were observed (red arrows in D). (E,F) Calbindin-labeling shows less well organized mossy fibers in the KO section (F) compared to an equivalent WT section (E). Red arrowheads indicate a more fragmented appearance of fibers in the KO. Only strongly CB-labeled KO sections were used in this analysis. (G–K) Golgi-Cox labeling shows the association of interneuron-like cells (red arrows) with CA3 pyramidal cells, even those displaced in a separate upper pyramidal layer in the KO (H). Pyramidal cells (blue, stained with Nissl) were present in 3 approximate layers in this KO animal (2 are observed here labeled 1, 2 in H). (I–K) The three cells shown in H are shown in better focus individually in I–K. These cells show the typical morphology of CA3 basket cell or axo-axonic-like interneurons [14], with fusiform cell bodies, and one or two dendrites originating from the apical pole, which then branch proximally to give radially oriented dendrites in the stratum radiatum. In addition, such cells have several basal, spine-free dendrites, branched close to the cell body and extended toward the alveus. Scale bars: A (for A, B), 500 µm; and C (for C, D); E (for E, F); G (for G, H), 50 µm; I (for I, J, K), 44 µm. dg, dentate gyrus; fi, fimbria; so, stratum oriens.

The *Dcx* KO hippocampal CA3 region is divided into several distinct pyramidal cell layers (Figure 1A, B). We questioned here the innervation of this region by the dentate gyrus (DG), and its association with interneurons, in order to better understand the potential functional consequences of such defects.

Histological analyses of cresyl violet-stained sections of 5 KO and 3 wild type (WT) adult brains at age 4 months on the C57BL/6 (B6) background, revealed a very similar organization of the DG between the genotypes (Figure 1A–D). Our previous studies also showed no major differences in the development of the DG at late embryonic and neonatal stages [8]. However, in KO hippocampi, CA3 cells were abnormally dispersed in the CA3b and CA3c regions, between the fimbria and the entrance to the DG, in at least two distinct layers (Figure 1D, H), compared with the single diffuse layer of WT hippocampi (Figure 1C). Such abnormalities were observed at all levels in the hippocampus along the rostro-caudal axis. CB-labeled mossy fibers, the axons of DG cells, appeared correspondingly less well-organized in the CA3 region of KO hippocampi (Figure 1E, F, WT, n = 9; KO, n = 6). Mossy fibers may therefore innervate abnormally dispersed CA3 pyramidal cells in a spatially appropriate fashion in KO mice. In addition, interneuron-like cells, identified by their morphology [14] using Golgi staining, were also observed in close proximity to pyramidal cells in each of the KO CA3 layers (Figure 1G–K, Figure S1). Altogether, these data suggest that mossy fibers and interneurons adapt their innervation patterns to target abnormally dispersed CA3 cells.

### Adult *Dcx* KO Mice Weigh Less than WT Mice and Are Hyperactive

**Figure 2:**
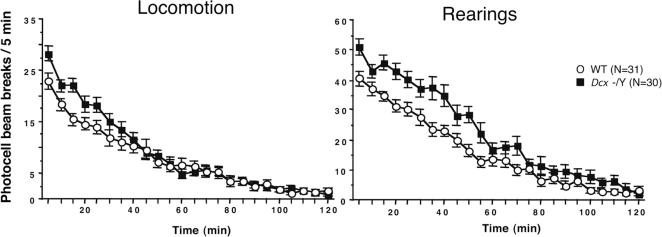
Dcx KO Mice are Hyperactive. Time-course of mean±SEM spontaneous horizontal (locomotion) and vertical (rearings) behavioural activity in WT (n = 31) and KO (n = 30) mice. Adult mice (aged 5–6 months) were introduced individually in activity boxes and automatically recorded over a 2 h period. An ANOVA test revealed significance between the 2 strains for horizontal activity, (genotype, *F*_1,1416_ = 14.48, *P*<0.0001; time, *F*_23,1416_ = 80.82, *P*<0.0001 and genotype×time interaction, *F*_23,1416_ = 2.78, *P*<0.01); and for vertical activity, (genotype, *F*_1,1416_ = 102.71, *P*<0.0001; time, *F*_23,1416_ = 76.41, *P*<0.0001 and genotype×time interaction, *F*_23,1416_ = 1.785, *P*<0.01).

At weaning (4 weeks), there were no differences in body weight between KO (n = 35) and WT (n = 42) mice (respectively 11.2±0.42 and 11.7±0.41 g). However, at 4 months of age, the body weight of *Dcx* KO mice (n = 45) was significantly lower than WT littermates (n = 47), (respectively 28.9±0.4 and 30.8±0.4 g; ANOVA test, *P<*0.005, *F*_1,90_ = 11.18). We also observed that exposure to the novel environment of actimeter cages triggered a significantly higher level of spontaneous horizontal and vertical activity in KO adult mice (5–6 months of age) compared with their WT littermates (WT n = 31; KO n = 30; ANOVA test, Figure 2). This novelty-induced hyperactivity was also observed in younger animals (data not shown).

### *Dcx* KO Mice Exhibit Spontaneous Seizures and Show Changes in Seizure Sensitive Markers

Occasional spontaneous seizures were observed in *Dcx* KO mice (n = 5), characterized by abnormal head movements, rearing, and clonus of the forelimbs. No WT littermates showed similar behaviors.

The expression of various markers has been shown to be altered in the hippocampus after seizure activity induced by lesions, drug treatment, direct electrical stimulation and gene inactivation [15]–[22]. We therefore examined the expression of CB and NPY, known to be modified in such models of recurrent seizures, in untreated WT (n = 13) and KO (n = 16) mice of different ages (Table 1).

**Table 1:** Modification in Calbindin and NPY Expression in *Dcx* KO Mice.

Number of mice tested	Genotype	Age (months)	Number of mice with modifications	Calbindin	Neuropeptide Y
3	WT	3	0	-	-
3	KO	3	1	++	++
4	WT	6–7	0	-	-
5	KO	6–7	3	+/+++	+++/nd
1	WT	9	0	-	-
1	KO	9	0	-	-
3	WT	11–13	0	-	-
3(*)	KO	11–13	3	+++/++*	+++
2	WT	15–18	0	-	-
4	KO	15–18	2	++/+	+++
KO mice showing changes			9 (56.3%)		

A subset of *Dcx* KO mice show correlated calbindin and NPY expression changes potentially related to spontaneous seizures. WT: wild-type; KO: knockout (female and male); +: slight modifications; ++: moderate modifications; +++: strong modifications; nd: not determined; ^*^: mouse showing spontaneous epileptic seizures.

**Figure 3:**
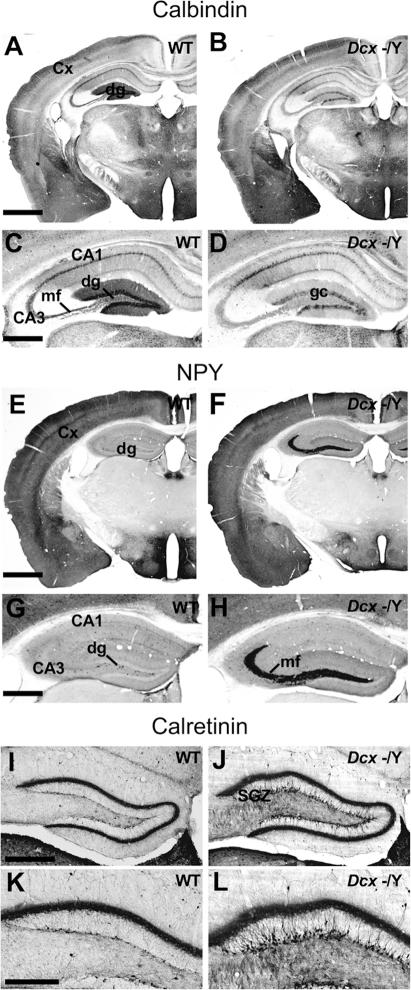
Untreated KO Mice Show Changes in Seizure Sensitive Marker Expression. (A–D) CB immunoreactivity in an untreated WT mouse (A,C) and a KO mouse (B,D), sacrificed at 7 months of age. In the hippocampus of the KO mouse, the expression of CB is reduced in the dg cell layer and dendrites, and in mossy fibers (D). Although the corpus callosum appears thinner in the KO mouse section, this is not typical for *Dcx* KO mice on the C57BL/6 background (8). (E–H) NPY immunoreactivity in an untreated WT mouse (E,G) and a KO mouse (F,H), sacrificed at 7 months of age. High magnification of the hippocampus shows NPY neoexpression in the mossy fibers of the dg in the KO mouse (H). Ectopic NPY accumulates in the mossy fibers of dentate granule cells probably because it is transported through the fibers to the terminals, from which it can be released [61]. No obvious differences in the expression of NPY and CB were observed in other brain structures, as previously reported by others [19]. (I–L) Calretinin immunoreactivity in an untreated WT mouse (I,K) and a KO mouse (J,L). Note the disorganization and increase in expression and number of calretinin-labeled cells in the subgranular zone (SGZ) in KO sections. Analysis of 9 KO animals showed modifications in the SGZ in 7 of them (see also Figure S2), although 3 of these animals showed only a minor disorganization of the SGZ cells. The granular layer itself showed no obvious differences between the genotypes. Cx: cortex; dg: dentate gyrus; gc: granular cells; mf: mossy fibers; SGZ: subgranular zone. Scale bars: A (for A, B, E, F), 1 mm; C (for C, D, G, H), 200 µm; I (for I, J), 300 µm; K (for K, L), 100 µm.

CB is present in subpopulations of interneurons, pyramidal and DG cells in the isocortex and hippocampus and the occurrence of seizures is often associated with a decrease in its expression, particularly in the DG [20], [23]. In the hippocampi of WT mice (aged 3–18 months) we therefore observed CB expression in the cell bodies and dendrites of DG cells, as well as in the mossy fibers projecting to the CA3 region (Figure 1E,F; 3A,C). Sparse CB-positive interneurons were observed in the stratum radiatum /lacunosum moleculare region of the CA3 area. In 9/16 *Dcx* KO animals tested (56.3 %), we observed a clearly reduced intensity of CB staining in the dendrites of DG cells, their cell bodies and mossy fibers, throughout the rostro-caudal axis of the hippocampus, compared to labeling in WT mice (Figure 3A–D, Table 1).

By contrast, hippocampal NPY expression has been shown to be upregulated after seizures in epileptic animals, possibly reflecting an endogenous mechanism that opposes excessive hippocampal excitability [21]. In particular, a neoexpression of this neuropeptide in DG cell mossy fibers has been reported in epileptic animals [22]. We therefore examined this marker in adjacent slices to those labeled for CB. A strong ectopic expression of NPY was observed in mossy fibers stemming from the DG (Figure 3E–H) in the same KO mice showing a reduced CB expression. Such a modification was not observed in other *Dcx* KO mice with a normal expression of CB, nor in WT mice.

In one mouse where spontaneous epileptic seizures were previously observed and which was sacrificed at 11 months, a clear reduction of CB expression and a dramatic increase in NPY expression were detected (Table 1). In addition, KO mice as young as 3 months were shown to have a changed expression of these markers whereas none of the WT mice (n = 13) showed these changes, even at 15–18 months (n = 2). These combined modifications of CB and NPY expression were therefore observed in 9 out of 16 KO mice (56.3 %) tested (Table 1). This proportion may however be underestimated, since in other models such alterations have been shown to occur with a delay after seizure onset, and the duration of such changes is also unknown [20], [23]. Altogether, the combined results of our CB and NPY labelings, and the previous association of these markers with epilepsy [20]–[23], suggest, albeit indirectly, that a large proportion of *Dcx* KO mice may have experienced recurrent spontaneous epileptic seizures.

Certain mice (9 KO, 4 WT) were also tested for calretinin expression, which labels newly generated neurons of the hippocampal subgranular zone, as well as interneurons (Figure 3I, K). Four KO animals showed a moderate to extensive increase in the number and intensity of labeled subgranular zone neurons (Figure 3J, L), with 3/4 of these animals also showing changes in NPY/CB (Table S1). Such modifications of calretinin expression have previously been correlated with decreased CB expression and epilepsy onset in other models [20]. Three other KO animals showed a minor disorganization of such cells (Figure S2), often affecting the supragranular blade, as we previously reported [12], with 2/3 animals also showing changes in NPY/CB. The remaining 2 KO animals and the 4 WT mice tested showed no obvious abnormalities in calretinin, CB or NPY. Thus it appears that changes in calretinin expression can be variable and are not always strictly correlated with changes in CB and NPY. Indeed, the minor disorganization of cells observed in some animals may represent a separate phenomenon, unrelated to the increased number of cells observed in other animals. However, Ruttimann et al. [20] showed a short transient change in expression of calretinin, compared to longer term changes for CB in epileptic GABA B1 -/- mutant mice and this may partly explain the variability observed here.

### *Dcx* KO Mice Are More Susceptible to Chemoconvulsants than WT Mice

To further characterize seizure susceptibility in *Dcx* KO mice, we first attempted to induce audiogenic seizures. WT (n = 8) and KO (n = 8) animals, aged 28–33 days [24], were exposed to a 90 decibel sound for 90 sec in two tests separated by 48 h. Wild running was observed in one WT animal, but no tonic or tonic-clonic seizures occurred in either WT or KO animals (data not shown). Similarly, the sensitivity to reflex-seizures induced by tactile stimulation was explored in 2 month old WT (n = 10) and KO (n = 10) animals. No behavioral arrest, myoclonic jerks or clonic seizures were observed in either group (data not shown).

We then tested the response of KO animals to PTZ and KA. Doses were adjusted from previous reports [25], [26] and behavior was subsequently assessed by two investigators blind to the genotypes. Following PTZ injections (30 or 35 mg/kg, i.p.) in 3–6 month old WT (n = 10) and KO (n = 10) animals, clonic or tonic seizures were observed in 7 KO mice compared to only 1 WT animal (exp. B and C, Table 2), suggesting an enhanced susceptibility to PTZ-induced seizures in KO animals.

**Table 2:** Increased Susceptibility of *Dcx* KO Mice to PTZ-induced Seizures.

PTZ doses	Genotype	n	*0*	*1*	Response *2*	***3***	Death
**A**: 50 mg/kg	WT	8	1	0	2	1	5
behavior	KO	8	0	3	0	4	3
**B**: 35 mg/kg	WT	4	3	0	0	1	1
Behavior	KO	4	0	0	2	2	1
**C**: 30 mg/kg	WT	6	6	0	0	0	0
behavior	KO	6	0	3	2	1	0
**D**: 30 mg/kg	WT	3	3	0	0	0	0
EEG (table 3)	KO	7	2	2	0	3	2
Sum of	WT	13	12	0	0	1	1
**B–D**	KO	17	2	5	4	6	3

***0***, no response; ***1***, myoclonic jerks; ***2***, clonic seizures associated with trembling and chewing; ***3***, tonic-clonic seizures associated with extension of fore- and hind limbs with animals falling on its side. Death, which could occur without behavioral seizures, was noted independently of the seizure score. We observed here that whereas the convulsive dose 50 s (CD50s) of 59.13 mg/kg was previously reported in C57BL/6J mice, PTZ at 50 mg/kg i.p. induced death in a large proportion of our KO and WT mice within less than 5 min following injection (C57BL/6N background, exp **A**). Results obtained for the EEG experiment (**D**) are shown separately to the data obtained at the same concentration for the behavioral assessment alone (**C**), to take into account the implantation of the electrodes and the different sites where these experiments were performed.

When PTZ (30 mg/kg, i.p.) was injected in mice equipped with hippocampal and cortical electrodes (exp D, Table 2; n = 3 WT and n = 7 KO), no behavioral seizures or EEG abnormalities were observed in WT animals during 10 min following injection (Table S2). In 2 KO mice, PTZ induced a clonic-tonic seizure within 3 min, rapidly followed by death. In another KO mouse, hippocampal spikes (4–5/min) occurred within 3 min and were followed by a discharge of spikes and spike-and-waves associated with a clonic seizure (latency = 8.5 min; duration = 24 sec). Myoclonic jerks were observed in 2 further KO animals, although no changes were observed in EEG activity. No overt changes of either behavior or EEG activity were observed in the 2 remaining KO mice.

**Figure 4:**
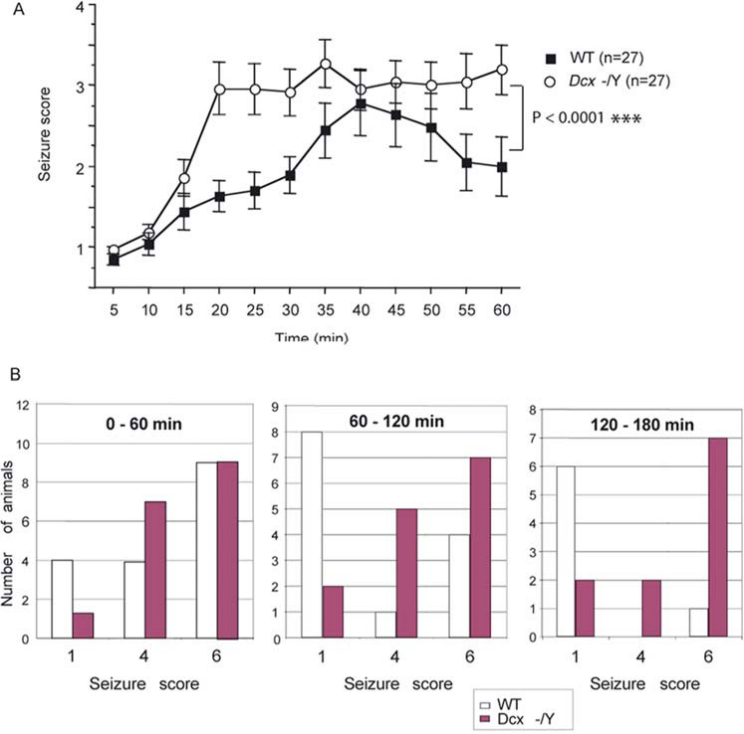
*Dcx* KO Mice are More Susceptible to KA-induced Seizures. (A) In the first 60 min after KA injection, a significant increase in the progression of seizure-related behavior was observed in KO mice (white circles), compared to WT (black squares). ANOVA: genotype, *F*_1,1565_ = 37.65, *P*<0.0001; time, *F*_11,565_ = 12.5, *P*<0.0001 and genotype×time interaction, *F*_11,565_ = 1.4, *P*>0.05. KO mice were scored significantly higher at 15 min post-injection than WT mice and the increased severity of their reaction to KA continued throughout the initial 1 h monitoring period. *** differs from control at P<0.0001. (B) Bar histograms show that, during the three hour monitoring period, more KO mice show rearing and falling (score 4) or progression to severe tonic, clonic seizures (score 6) compared to WT mice. Inversely, more WT mice show less severe behavior (1, immobility). Thus, KO mice are significantly more susceptible to KA-induced seizures than WT mice. Seizures were rated according to a previously defined scale [59]: 1: immobility; 2: forelimb and/or tail extension; 3: repetitive movements, head bobbing; 4: rearing and falling; 5: continuous rearing and falling: 6: severe tonic-clonic seizures.

KA (20 mg/kg, i.p.) was injected in a different series of WT (n = 27) and littermate KO (n = 27) animals, aged 3–6 months. During the first hour following injection, 13 WT animals became immobile or displayed stereotypic movements (behavioral scores rated 1, 2 or 3, Table S3), whereas the 14 others showed convulsions or severe seizures (scores 4, 5 or 6). In contrast, only 8 KO mice were rated 1–3 in this test compared to 19 KO animals showing convulsions or severe seizures (Figure 4A, B; Table S3). Furthermore, KO mice were scored significantly higher than WT in the first 15 min following KA injection, as well as throughout the 3h monitoring period (Figure 4B). In both groups, several mice died in the week following the KA test (n = 12 KO, 11 WT), with 4 KO compared to 0 WT mice dying in the first 40 min.

When KA (20 mg/kg, i.p.) was administered in animals equipped with hippocampal and cortical electrodes (n = 5 WT; n = 4 KO), discharges of spikes associated with behavioral arrest were observed in the hippocampus and cortex in both groups. These discharges were similar in number, latency and mean duration during the first hour post-injection (Table 3). However, all KO mice displayed forelimb clonic seizures associated with a discharge of spikes and polyspikes within the first 60 min post-KA whereas this was observed in only 2 WT mice. These convulsive seizures were significantly more frequent, and with a shorter latency and longer mean duration during the first hour post KA in KO compared to WT mice (Table 3).

**Table 3:** Summary of EEG Results within 1 Hour Following KA Injection.

	WT	KO
	n = 5	n = 4
Latency of first non convulsive discharge (min)	26.00±4.95	24.00±8.76
Number of non convulsive discharges	2.80±0.97	2.00±0.91
Mean duration of non convulsive discharge (sec)	22.02±5.78	25.96±13.19
Latency of first convulsive seizure (min)	106.00±33.92	28.50±8.88∇
Number of convulsive seizures	0.80±0.58	3.50±1.04*
Mean duration of convulsive seizure (sec)	19.53±12.12	46.33±6.43

Mean±SEM of latency, number and mean duration of non-convulsive discharges and clonic convulsive seizures recorded by hippocampal and cortical EEG in WT and *Dcx* KO mice during the first hour following the injection of KA (20 mg/kg, i.p.). Mann-Whitney tests, ^*^ z = 2.01, *P*<0.05. Latency: ∇, P = 0.08.

These data clearly show that *Dcx* KO mice are more prone to seizures induced by convulsants, with both PTZ and KA inducing convulsive seizures more readily in KO animals, than WT littermates.

### Video-EEG Recordings Reveal Ictal Activity and the Initiation of Spontaneous Seizures in the Hippocampus

Our data converge to suggest that *Dcx* KO mice are epileptic. Indeed, spontaneous convulsive seizures were observed occasionally in KO mice, expression of seizure-sensitive markers in the hippocampus was abnormal and these animals showed an increased sensitivity to chemo-convulsants. We performed video-EEG monitoring in an attempt to detect spontaneous electrographical and behavioural seizures in the absence of convulsant and to characterize associated behaviors. Animals (6 WT, 5 KO, aged 5–6 months) were regularly recorded for periods of 12h during the day time. EEG recordings showed occasional hippocampal spikes and short periods of theta oscillations associated with exploratory behavior in both WT and KO animals. Spectral analyses of the EEG activity during rest did not reveal any significant differences between the two lines (data not shown).

**Figure 5:**
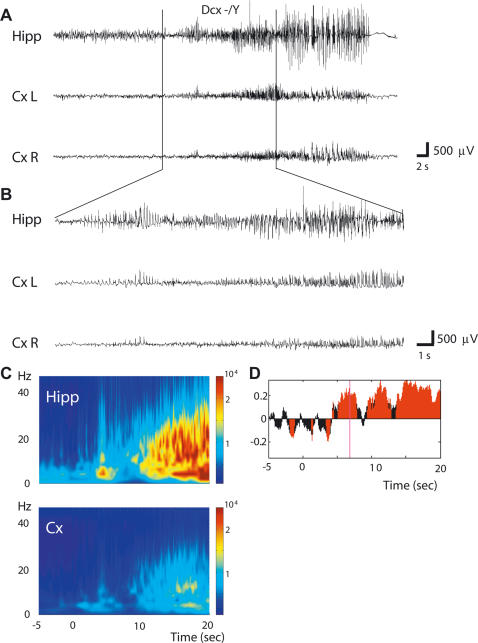
Spontaneous Clonic Seizures in *Dcx* KO Mice. (A) Typical example of hippocampal and cortical EEG recordings of a spontaneous seizure observed in a *Dcx* KO mouse associated with rearing, head nodding and forelimb clonus. (B) EEG recording at lower speed of the same seizure suggesting initiation in the hippocampus. Hipp: right hippocampus; Cx L: left cortex; Cx R: right cortex. (C) Averaged (n = 5 spontaneous seizures) time–frequency chart of signal power 5 sec before and 20 sec after the onset of the seizure in the hippocampus (Hipp) and the cortex (Cx). Hz, hertz. (D) Difference of GPDC (averaged over 5 seizures) between hippocampal and cortical recordings. A positive value indicates a direction of information transfer from hippocampus towards cortex. Red areas indicate a significant GPDC difference (p<0.005). Statistics were obtained using surrogate data in which phase relationships were destroyed by phase randomization of frequency spectra.

Spontaneous seizures were observed in 2 of 5 KO mice and a total of 5 seizures were recorded (Figure 5, Video S1). These seizures occurred when the animal was at rest or during slow wave sleep and always started with a slight movement of the head immediately followed by stereotyped sniffing associated with the first spikes in the hippocampus. As the discharge developed and the amplitude increased, the animal reared and displayed head nodding and clonus of the forelimbs. The discharge lasted between 28 and 40 sec after which flattening of the EEG was observed (Figure 5A). During this post-ictal EEG depression, forelimb clonus could still be observed for about 1 min. Following this phase the animals remained immobile for 2–3 min after which they generally groomed. No running, jumping or tonic phases were observed during these analyses. Examination of EEG recordings at lower speed suggested that the seizures were initiated in the hippocampus and then propagated to the cortex (Figure 5B). This was confirmed by time-frequency analyses in the 2–48 Hz range showing a higher power of the EEG signal recorded in the hippocampus as compared to the cortex (Figure 5C). In addition, estimation of the direction of information transfer [27] indicated that the hippocampus mainly leads the cortex at least during the first 20 sec of the seizures (Figure 5D).

These data hence better characterize and confirm electrographically the occurrence of spontaneous clonic seizures in the absence of convulsant in *Dcx* KO mice and localize seizure onset to the hippocampus, the site of visible morphological abnormalities.

### Enhanced Excitability in the CA3 Region of *Dcx* KO Mice

**Figure 6:**
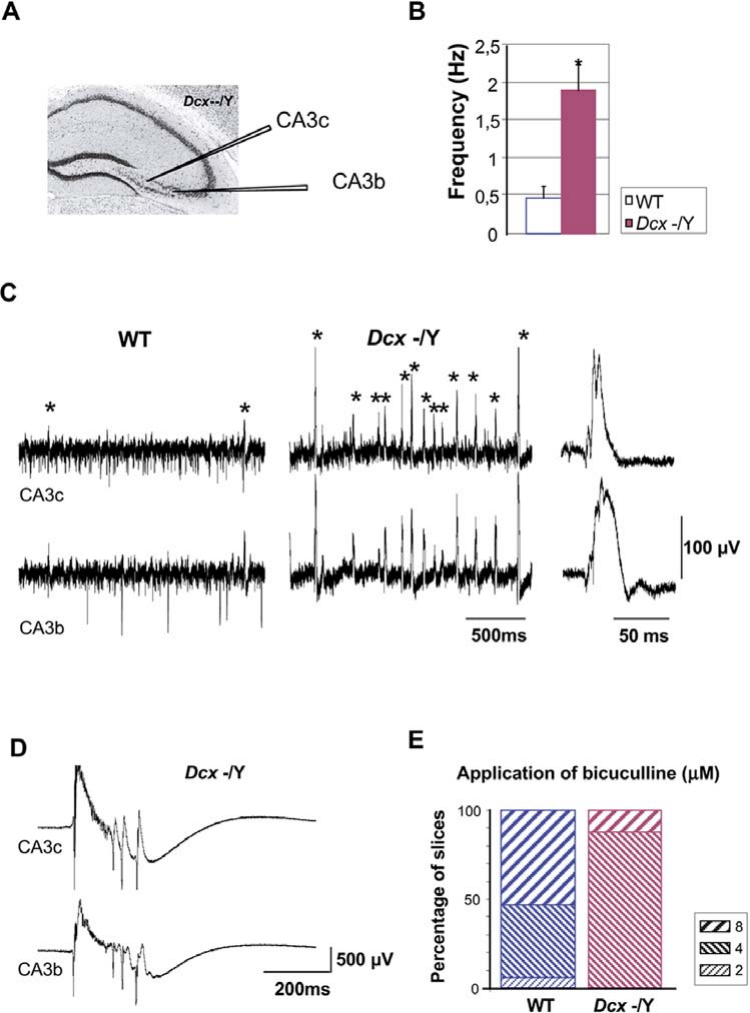
Enhanced Excitability and Lower Threshold for Epileptical Events in *Dcx* KO Hippocampal Slices. (A) Position of the two tungsten electrodes placed in the stratum pyramidale of the CA3b and CA3c regions. (B) Bar graph shows a higher frequency of the synchronously generated sharp wave-like events in KO slices than in WT (1.91±0.34 Hz, KO, n = 10 vs. 0.48±0.15 Hz, WT, n = 11, Students's t-test, P<0.001). Error bars indicate SEM. (C) Extracellular recordings of synchronously generated sharp wave-like activities in the CA3b and CA3c regions from WT (left) and KO (center) animals. * indicates synchronous events, which are seen in both CA3b and c regions. Right panel shows 1 synchronous sharp wave-like activity in KO slices. (D) Interictal bursts initiated in the CA3 region on exposure to bicuculline at 4 µM in KO hippocampal slices. (E) Bar graph shows the percentage of slices that reached bursting threshold in bicuculline from 2 to 8 µM. KO mice are predisposed to epileptiform activity with moderate bicuculline application.

In order to test for functional changes in hippocampal circuitry which might explain the indications of epileptic activity, hippocampal slices were prepared (n = 11 WT, n = 10 KO) and extracellular signals recorded from the stratum pyramidale of the CA3b and CA3c regions (Figure 6A). Electrodes were placed with a separation of 500 µm to detect the activity of distinct CA3 neuron populations [28].

Spontaneous recurring population oscillations similar to sharp waves observed *in vitro* [29] were generated in the CA3b and CA3c regions from both WT and KO slices. This sharp wave activity is similar to EEG activity generated in sleeping and resting animals under normal physiological conditions. Each wave was evident as a positive-going field potential with a sharp rise and a slower decay (Figure 6C). Synchronous sharp wave-like events were however, found to occur more frequently in *Dcx* KO slices (n = 10, 1.91±0.34 Hz) than WT slices (n = 11, 0.48±0.15 Hz, Students t-test, P<0.001, Figure 6B and C). These results therefore show an enhanced synchronous population activity in the *Dcx* KO CA3 region. Such activity might reflect an enhanced cellular excitability or recurrent synaptic connectivity, both of which should favor hippocampal epileptiform activity.

### *Dcx* KO Mice Show a Lower Threshold for Epileptiform Activity Induced by Bicuculline

Bicuculline, a GABA_A_ receptor antagonist, induces interictal-like epileptiform events [30], characterized by high frequency oscillations (150–300 Hz) superimposed on an underlying negative field potential shift (Figure 6D). We compared the threshold dose for the induction of these events in WT and *Dcx* KO hippocampal slices. Slices were exposed sequentially to a series of concentrations of bicuculline in the range 2–8 µM. Interictal bursts (Figure 6D) were initiated in the CA3 region on exposure to 4 µM bicuculline in 87.5 % of KO slices (n = 14/16), and in the remaining 12.5 % at 8 µM bicuculline (n = 2/16, Figure 6E). In contrast, only 41% of WT slices reached bursting threshold with 4 µM bicuculline (n = 7/17), with a further 53% (n = 9/17) at the higher concentration of 8 µM. Thus, with the exception of one WT slice bursting at 2 µM, KO slices showed a lower threshold for interictal activity than WT slices. The frequency of interictal-like epileptiform events did not differ significantly in KO (0.24±0.03 Hz) and WT (0.19±0.03 Hz) slices.

These data show that KO hippocampal slices have an enhanced excitability and lower threshold for the induction of epileptiform events, compared to slices from WT littermates. Combined with the anatomical data, behavioral and EEG analyses, these results strongly support the dysplastic hippocampus as the site of original epileptogenic events in *Dcx* KO animals.

## Discussion

We previously showed that *Dcx* KO animals have a normally laminated isocortex, but they exhibit interneuron migration defects and a disorganized hippocampus. In the present study, we set out to determine the consequences of these defects. We show that KO animals are hyperactive, and exhibit signs of chronic epilepsy, abnormalities that were not previously identified in another *Dcx* KO model [11].

Hyperactivity was observed in *Dcx* KO mice during exploration of novel environments. It is not yet clear if this phenotype is linked to the epilepsy exhibited by these mice, to potential cognitive deficits, or to other factors unrelated to the hippocampus. Indeed, hyperactivity and additional sensorimotor abnormalities have been linked to hippocampal dysfunction [31]. Hyperactivity was thus identified following hippocampal lesions in C57BL/6 mice [32], and in mouse models of mental retardation associated with hippocampal-dependent cognitive defects [33], [34]. *Tuba*1a mutant mice, another lissencephaly model, are also hyperactive and exhibit prominent hippocampal lamination defects [35]. On the other hand, hyperactivity has also been linked to isocortical interneuron abnormalities [36]. It will thus be important to further explore whether this particular phenotype is directly related to hippocampal lamination defects and potential cognitive deficits in *Dcx* KO animals, or alternatively, to more subtle isocortical abnormalities.

We demonstrate here that *Dcx* KO mice develop epilepsy, with seizures initiated in the hippocampus. Indeed, we observed both behavioral features of limbic seizures, and facilitated seizure induction by KA and PTZ, which have both been shown to target limbic structures [37], [38]. In contrast, seizures were not triggered by audiogenic or tactile stimuli, known instead to involve brainstem structures [39]. The primary involvement of the hippocampus is supported by the higher frequency of spontaneous synchronous activities observed in the *Dcx* KO CA3 region *in vitro*, combined with a lower threshold for interictal-like activities in the presence of bicuculline, suggesting enhanced neuronal excitability or recurrent synaptic connectivity of CA3 pyramidal cells. In addition, our EEG recordings of spontaneous seizures, along with time-frequency and information transfer analyses suggest that the hippocampus is the primary epileptogenic focus in *Dcx* KO animals. These converging data strongly suggest that discrete hippocampal dysplasia gives rise to functional abnormalities which render these animals more prone to seizures.

*DCX* mutations in human cause a spectrum of abnormalities ranging from type I lissencephaly to subcortical laminar heterotopia (SCLH), which are associated with mental retardation and epilepsy, the latter frequently refractory to treatment [6]. In type I lissencephaly, EEG recordings, show high amplitude and high frequency discharges, due to the neocortical disorganization and smooth brain surface, where neurons are organized in similar orientations, leading to an amplified signal [40]. In SCLH, at the other end of the spectrum, the EEG pattern and clinical symptoms can be correlated with the thickness of the heterotopic band. Patients with thin bands show a later onset of seizures which originate mostly in the temporal lobe as shown by scalp EEG [41]. In some cases the discharges evolve into complex partial seizures [42]. On the other hand, patients with thick bands usually start epilepsy early in infancy with mostly generalized seizures, and interictal multifocal abnormalities are generally observed on scalp EEG [43]. However, in these latter cases it is possible that the multifocal abnormalities mask an initiation of seizures in the hippocampus. Indeed, in some patients stereotactic EEG recordings using depth electrodes have indicated a regional or focal seizure onset which can involve the temporal lobe [44]. Thus the epilepsy observed in *Dcx* KO mice, with seizures initiated in the hippocampus and propagated to the cortex, is somewhat reminiscent of that observed in SCLH patients. However, it is also possible that the hippocampus is not the only structure generating seizures in such patients, and the human disorder is obviously more complex than the mouse model. To our knowledge, there are no clinical reports of isolated hippocampal lamination defects in other human patients, although such subtle abnormalities may be difficult to identify.

Other mouse models associating a susceptibility to epilepsy with neuronal lamination defects, such as *p35*, *Lis*1 and *reeler* mutant mice, show severe hippocampal pyramidal cell defects affecting both the CA1 and CA3 regions [45]–[47]. Notably *p35* and *Lis*1 KO mice exhibit spontaneous tonic-clonic seizures, whilst *reeler* mice have a reduced threshold for electroshock-induced seizures [48]. A defect in the hippocampal pyramidal cell layer is thus a common pathological feature of these mice. However, the DG and the isocortex are also abnormally organized, making it difficult to determine the primary causes of the epilepsy. In this respect, the *Dcx* KO mouse is unique, exhibiting subtle modifications of the hippocampal circuitry, associated with the susceptibility to epilepsy. No signs of hippocampal sclerosis (e.g. cell loss in hilar, CA1 or CA3 areas) or granule cell dispersion [49] were obvious in *Dcx* KO mice and the DG appears to form normally [8]. Therefore, with stable abnormalities in lamination largely restricted to the hippocampus proper, in particular the CA3 region, *Dcx* KO mice provide a pertinent model to further investigate the cellular mechanisms involved in seizure initiation.

Although controversial, seizure activity may influence dentate granule cell neurogenesis [50]–[53]. Our data, showing in certain animals an increase in both the number of calretinin-positive neurons and in the intensity of their labeling, point to increased neurogenesis in the dentate gyrus of certain *Dcx* KO mice. Since these newly generated neurons were often also disorganized, they could in addition contribute to network hyperexcitability and possibly promote subsequent seizures [54]. Further studies assessing neurogenesis in *Dcx* KO animals and its relationship to epileptic seizures are therefore warranted.

We show here that epileptiform activity is likely to be initiated in the CA3 region at or near the pyramidal cell defects. Interestingly CB-labeled mossy fibers are disorganized, suggesting that ectopic pyramidal cells still receive mossy fiber innervation. In addition, interneuron-like cells were identified in close proximity to dispersed, as well as correctly localized CA3 cells. These data therefore suggest that, following the malpositioning of neurons due to disrupted neuronal migration, spatially specific synaptic projections could still form contacts with ectopic cells. However, it remains to be determined whether such connections are formed appropriately and how synaptic transmission differs from that in WT animals. These questions need to be addressed to better understand the mechanisms underlying seizure initiation.

In summary, we describe here the first functional consequences associated with discrete hippocampal lamination defects. Importantly, we demonstrate that *Dcx* KO mice have an epileptic phenotype and suggest that disrupted neuronal migration results in enhanced seizure susceptibility similar to the human condition. A further characterization of the consequences of the lamination defects and possible interneuron abnormalities will help to better characterize the pathogenic mechanisms at play. The *Dcx* KO model will thus permit further studies of how abnormal neuron position leads to possible changes in cell excitability or connectivity resulting in epileptic synchrony. Such studies should shed light on the pathophysiology of cortical migration disorders associated with refractory epilepsy, and may in the future allow the development of novel therapies.

## Materials and Methods

### Mice

*Dcx* mutant mice were maintained on the C57BL/6N (B6) background after more than 10 generations of backcrosses and were produced and genotyped as previously described [12]. Experiments were conducted during the light phase of a 12 h light/dark schedule, with lights on at 07:30 a.m. All behavioral and pharmacological experiments were performed on 1–6 months old males. The studies were performed in accordance with the European Communities Council Directive (86/809/EEC) regarding the care and use of animals for experimental procedures and approved by local ethical committees.

### Immunohistochemistry and Golgi-Cox staining

Immunohistochemistry was performed as described previously [12]. Markers used with immunoperoxidase detection were anti-calretinin (1∶10000, rabbit; Swant laboratories, Switzerland); anti-NPY (1∶10000, rabbit; Sigma, St. Louis, MO) and anti-CB (1∶20000, rabbit; Swant laboratories, Switzerland).

Golgi-Cox staining was performed as described by Gibb and Kolb [55]. WT and KO adult mice (6 months) were anesthetized with pentobarbital (Ceva Santé Animale; 0.15 ml/ 10g body weight) and perfuzed intracardially with 9% (w/v) saline buffer, and brains were placed in Golgi-Cox solution [56] at room temperature in the dark for 10 days. Brains were then placed in 30% (w/v) sucrose for 5 days at room temperature in the dark, prior to sectioning in 6% (w/v) sucrose using a vibratome (200 µm, VT 1000S, Leica, Germany). Sections were incubated in the dark firstly in NH_4_OH for 30 min, then after rinsing, in Kodak fixative for 30 min, and then were counter-stained with toluidine blue.

### Locomotor activity

Horizontal (locomotion) and vertical (rearing) activities of naive animals in novel environments were measured individually in plexiglass cages (20×15×25 cm), with automatic monitoring of photocell beam breaks every 5 min for 2 consecutive hours (Imetronic, France). For horizontal activity, consecutive beam breaks were recorded from two separate photocell beams. The number of beam breaks in this case can be correlated with the walking distance (cm) and provides a direct index of locomotion.

### Behavioral assessment of seizure activity

#### Handling-induced seizures

Handling-induced seizure susceptibility in both WT and *Dcx* KO animals was assessed according to Todorova [57]. The handling stimulus involved lifting each mouse by the tail for 30 s, and moving it to a new cage. Each animal was observed for 30 s on 5 consecutive days.

#### Audiogenic seizures (AS)

Since AS susceptibility is age dependent [24], all animals were tested prior to weaning, (at 28-33 days) in a plexiglass cage (45×45 cm) covered by a grid. After 15 sec of habituation, animals were exposed to a computer-generated sound of 13 kHz at 90 decibels for 90 sec. Mice that did not display a seizure response were re-exposed 48h later and tested for sensitization-dependent audiogenic seizures. The intensity of the response pattern was rated simultaneously by two observers blind to the genotypes: 0, no response; 1, wild running; 2, clonic seizures and/or tonic flexion and extension; 3, death.

### Chemically induced seizures

#### Drugs

Pentylenetetrazole (PTZ, 30–50 mg/kg, Sigma, St Louis, USA) and kainate (KA, 15–20 mg/kg, OPIKA-1™, Ocean Produce International) were dissolved in 0.9% NaCl on the day of test and were injected intraperitoneally (i.p.) in a volume of 0.1 ml /10 g.

#### Induction and scoring of seizures

Chemically-induced convulsions were induced after an initial 2h period of habituation to the test cage, by i.p. injection of either PTZ, at the doses of 30, 35 and 50 mg/kg, or KA (15 or 20 mg/kg i.p.). The animals were immediately placed back into the test cages and their behavior and seizure-related activities were observed by two experimenters blind to the genotypes.

Following PTZ injections, scoring of seizures [58] was performed every minute for 10 min after injection as follows: 0, no response; 1, myoclonic jerks; 2, clonic seizures associated with trembling and chewing; 3, tonic-clonic seizures associated with extension of fore- and hind limbs with animals falling on its side. Death, which could occur without behavioral seizures, was noted independently of the seizure score. In our PTZ experiments, we observed that whereas the convulsive dose 50 s (CD50s) of 59.13 mg/kg was previously reported in C57BL/6J mice, PTZ at 50mg/kg i.p. induced death in a large proportion of our KO and WT mice within less than 5 min following injection (C57BL/6N background, exp A, Table 2).

For KA, mice were monitored for 30 seconds every 5 minutes during the first hour after the injection and every 20 minutes during the following 2 hours. Seizures were rated according to a previously defined scale [59] 1, immobility; 2, forelimb and/or tail extension; 3, repetitive movements, head bobbing; 4, rearing and falling; 5, continuous rearing and falling; 6, severe tonic-clonic seizures, death (after seizures).

#### Video- EEG recordings

Mice were anaesthetized (chloral hydrate, 0.4 g/kg, i.p.) and implanted with cortical and hippocampal electrodes as previously described [49]. After at least 2 weeks of recovery, video-EEG activity was recorded using a digital acquisition computer-based system (Coherence, Deltamed, France, sampling rate 256 Hz) while the mice were freely moving in a Faraday cage, as in previous studies [49]. When mice were examined for spontaneous seizures, their EEG activity was recorded concomitantly with their behavior (synchronized video) for up to 12 hours during the day, on 6–7 different days during a period of 6 weeks. To test convulsants, the animals were first recorded for 5 or 10 min before the PTZ or KA injection, respectively, and then for 10 min (PTZ) or 3 hours (KA) afterwards.

A time-frequency analysis of the spontaneous seizures in 2 KO mice was performed using an in-house developed toolbox of Statistical Parametric Mapping 5 software (www.fil.ion.ucl.ac.uk/spm, Wellcome Department of Imaging Neuroscience, University College London, UK) for dynamical analysis of intracerebral EEG. For each discharge, the amplitude (square-root of power) of oscillatory activity between 1 and 48 Hz, from 5 sec before the onset and up to 20 sec thereafter, was obtained using a standard time-frequency analysis based on Morlet wavelet transform [60]. For each frequency, the amplitude was computed on a 7 period length sliding time-window, providing an effective frequency specific time resolution. Time-frequency sampling of the time-frequency plane was 4 ms/2 Hz. The time-frequency plane was averaged over the 5 seizures.

To determine whether spontaneous seizures were initiated in the hippocampus or in the cortex, estimation of the direction of information transfer was performed using the generalized partial directed coherence (GPDC) test [27]. The GPDC was computed using the BioSig toolbox (http://biosig.sourceforge.net/) on a sliding time window (time width: 3 s; time step: 100 ms). The model order of autoregressive models used in GPDC was defined according to the Schwarz's Bayesian criterion.

Upon completion of the experiments, all mice were injected with a lethal dose of pentobarbital (Nembutal, 100 mg/kg, i. p.). Brains were frozen, and cut into 20-µm sections using a cryostat (CM 3050S, Leica, Germany). Histological analysis to determine electrode position was performed following cresyl violet staining.

#### Electrophysiology

Slices were cut at 380 µm from WT and KO mice (3–4 months) using a vibratome (HM 650V, Microm). 6 KO and 4 WT mice were used for the sharp wave experiments and 5 KO and 4 WT animals for the bicuculline experiments. Mice were anesthetized with 80 mg/kg of ketamine HCl/ Xylazine HCl solution (Sigma, St. Louis, MO). The brain was removed and chilled in ice-cold, oxygenated artificial cerebrospinal fluid (ACSF) of the following composition (in mM): 250 sucrose, 1 KCl, 26 NaHCO_3_, 10 D-glucose, 1 CaCl_2_, 10 MgCl_2_. Transverse slices were prepared from the level approximately two-thirds of the way down the septo-temporal arc and placed in an interface recording chamber [29]. Slices were perfused with an oxygenated ACSF containing (in mM): 124 NaCl, 4 KCl, 26 NaHCO_3_, 10 D-glucose, 2 CaCl_2_, 2 MgCl_2_ at 35°C, while their upper surface was exposed to a humidified 95% O_2_/ 5% CO_2_ (v/v) atmosphere. Slices were exposed sequentially for 20 min to a series of concentrations of bicuculline (Tocris, Ellisville, USA) in range of 2–8 µM.

Multi-unit activities were recorded with extracellular electrodes made from tungsten wire of 50 µm diameter (Phymep, Paris, France). Up to 3 electrodes were mounted on holders controlled by separate manipulators. Differences in potential between each tungsten electrode and a reference Ag-AgCl electrode were measured using a 4-channel amplifier (AM system, model 1700, Carlsborg WA, USA). Extracellular signals were amplified 1000 x and filtered with pass band between 1 Hz and 10 kHz. Signals were digitized at 10–20 kHz using a 12-bit, 16 channel analog-to-digital converter (Digidata 1200A, Axon Instruments), and visualized on a PC using the program Axoscope (Axon instruments).

#### Statistical analyses

The data were analyzed by unpaired Student's t-tests, and repeated measures of analysis of variance (ANOVA) to assess the interaction between genotypes (between factor) and time (within factor). When variables did not follow a normal distribution, statistical analyses were carried out using the nonparametric Mann-Whitney rank sum test. Only significant statistical tests are reported in the text, with the significance established at a *P*-value<0.05. Error bars represent s.e.m. The data presented in Figure 4B are raw data (descriptive statistics).

## Supporting Information

Table S1 Modification in Calbindin NPY and Calretinin Expression in Dcx KO Mice.
(0.04 MB DOC)

Table S2 Summary of EEG results within 10 min following PTZ injection.
(0.03 MB DOC)

Table S3 Susceptibility of Dcx KO and WT mice to KA (during 1 hour observation period).
(0.03 MB DOC)

Figure S1 A focal z series of interneuron-like cells shown in Fig 1H, compared to a CA3 pyramidal cell in the same hippocampal section. (A–E) The Golgi-Cox stainings were performed in 40 µm brain slices and the three soma of interneuron-like cells are not all in focus in the same plane. This focal series helps show that each cell shows the typical morphology of a CA3 basket cell or axo-axonic-like interneuron [14], with fusiform cell bodies located within or adjacent to the pyramidal cell layer, and one or two dendrites originating from the apical pole, which then branch proximally to give radially oriented dendrites in the stratum radiatum. In addition, such cells have several basal dendrites branched close to the cell body and extended toward the alveus. Spines are rarely present on these few branches. Despite this recognisable morphology, we cannot specifically say what type of interneurons these are, because of the absence of a labelled axon arbor. (F, G) CA3 pyramidal cells (arrow in F and same cell shown in G) differ from this because they have one prominent apical dendrite emerging from a triangular soma, and this is radially oriented in the stratum radiatum where it is branched into large diameter segments, and in the stratum lacunosum moleculare where they emit several branches. Basal dendrites are also numerous in the stratum oriens. In addition the dendritic tree is typically densely covered with spines. The proximal apical dendrite has large complex spines (the thorny excrescence, arrowhead G) which form complexes with the large mossy fiber terminal. Scale bar E (for A-E); F, 75 µm; G, 37.5 µm.
(17.83 MB TIF)

Figure S2 Changes observed in calretinin -positive cells in the subgranular zone of the dentate gyrus in Dcx KO animals. (A) Two KO animals were observed to have a greatly augmented number of disorganized calretinin-positive cells, as shown here for one animal. Both animals also showed changes in NPY and CB. (B, C) Two further KO animals showed increased and/or disorganized calretinin-positive cells. The animal in B also showed changed NPY and CB. (D, E) Certain KO animals (n = 3) showed subtly disorganized calretinin-positive cells which were however not visibly increased number. The animal shown in D did not show changes in NPY and CB, whereas two further animals (one of which is shown in E) did. (F) Two KO animals, one of which is shown here, showed no overt changes in calretinin-positive cells, and no changes in NPY and CB. (G) A WT section is shown for comparison. 4 WT animals were analyzed. Scale bar G (for A–G), 100 µm.
(5.83 MB TIF)

Video S1 Video-EEG of a *Dcx* KO mouse suffering a spontaneous seizure (http://neurosciences.ujf-grenoble.fr/equipes/equipe9/index.html). A spontaneous seizure is exhibited by a *Dcx* KO mice in channel 2. Initially the mouse is resting. The first behavioral signs of the seizure are agitation of the head, followed by rearing and clonus of the forelimbs. This is followed by immobility for a short period.
(2.36 MB MOV)
