# Supplementary material for: Epilepsy in Dcx Knockout Mice Associated with Discrete Lamination Defects and Enhanced Excitability in the Hippocampus
Source: PLoS One. 2008 Jun 25;3(6):e2473. doi: 10.1371/journal.pone.0002473 (PMC2429962; doi:10.1371/journal.pone.0002473)
Supplement: Table S1 — Modification in Calbindin NPY and Calretinin Expression in Dcx KO Mice. (0.04 MB DOC) [file pone.0002473.s001.doc]

**Table S1.** Modification in Calbindin NPY and Calretinin Expression in *Dcx* KO Mice.

| Mice tested | Genotype | Age  (months) | Calbindin | Neuropeptide Y | Calretinin |
| --- | --- | --- | --- | --- | --- |
| 140  143  185  165  183  184 | WT  WT  WT  KO  KO  KO | 3  3  3  3  3  3 | ≠  ≠  ≠  ≠  **-**  ≠ | ≠  ≠  ≠  ≠  **+**  ≠ | nd  nd  nd  ≠  **+**  **+** |
| 123  138  108  109  127  137  963  776  773 | WT  WT  KO  KO  KO  KO  WT  WT  KO | 6  6  6  6  6  6  6,5  7  7 | ≠  ≠  **-**  ≠  ≠  **-**  ≠  ≠  **-** | ≠  ≠  **+**  ≠  ≠  **+**  ≠  nd  nd | ≠  ≠  **+**  ds  ≠  **+**  nd  nd  nd |
| 998  001 | WT  KO | 9  9 | ≠  ≠ | ≠  ≠ | ≠  nd |
| 990  991  891  893  962  983 | KO  KO  WT  WT  WT  KO | 11  11  12.5  12.5  13  13 | **-**  **-**  ≠  ≠  ≠  **-** | **+**  **+**  ≠  ≠  ≠  **+** | ds  ds  nd  nd  ≠  nd |
| 892  842  846  841  793  775 | WT  KO  WT  KO  KO  KO | 15  16  18  18  18.5  18.5 | ≠  **-**  ≠  **-**  ≠  ≠ | ≠  **+**  ≠  **+**  ­≠  ≠ | nd  nd  nd  nd  nd  nd |

WT: wild-type; KO: knockout (female and male); ≠: no modifications; -: decreased expression; +: increased expression; ds: disorganization; nd: not determined.
