# Supplementary material for: Epilepsy in Dcx Knockout Mice Associated with Discrete Lamination Defects and Enhanced Excitability in the Hippocampus
Source: PLoS One. 2008 Jun 25;3(6):e2473. doi: 10.1371/journal.pone.0002473 (PMC2429962; doi:10.1371/journal.pone.0002473)
Supplement: Table S2 — Summary of EEG results within 10 min following PTZ injection. (0.03 MB DOC) [file pone.0002473.s002.doc]

**Table S2**. Summary of EEG results within 10 min following PTZ injection.

| Genotype | Mouse | Hippocampal  spike | Hippocampal  discharge | Behavior | Death |
| --- | --- | --- | --- | --- | --- |
| WT | 353  352  363 | 0  0  0 | 0  0  0 | Normal  Normal  Normal | 0  0  0 |
| KO | 362  371  356  372  373  354  364 | +  +  +  0  0  0  0 | +  +  +  0  0  0  0 | 3(tonic/clonic…)  3(tonic/clonic…)  3(tonic/clonic…)  1 (jerks)  1 (jerks)  0  0 | +  +  0  0  0  0  0 |
