# Supplementary material for: Epilepsy in Dcx Knockout Mice Associated with Discrete Lamination Defects and Enhanced Excitability in the Hippocampus
Source: PLoS One. 2008 Jun 25;3(6):e2473. doi: 10.1371/journal.pone.0002473 (PMC2429962; doi:10.1371/journal.pone.0002473)
Supplement: Table S3 — Susceptibility of Dcx KO and WT mice to KA (during 1 hour observation period). (0.03 MB DOC) [file pone.0002473.s003.doc]

**Table S3.** Susceptibility of *Dcx* KO and WT mice to KA (during 1 hour observation period).

| KA doses | Genotype | n | ***1*** | ***2*** | ***3*** | **Response**  ***4*** | ***5*** | ***6*** | death |
| --- | --- | --- | --- | --- | --- | --- | --- | --- | --- |
| 20mg/kg  behavior | WT  KO | 27  27 | 4  1 | 6  2 | 3  5 | 4  7 | 0  1 | 10  11 | 11  12 |
| 15mg/kg  behavior | WT  KO | 4  4 | 1  0 | 2  2 | 1  2 | 0  0 | 0  0 | 0  0 | 0  0 |

***1***, immobility; ***2***, forelimb and/or tail extension; ***3***, repetitive movements (clonies), head bobbing; ***4***, rearing and falling; ***5***, continuous rearing and falling; ***6***, severe tonic-clonic seizures.
