# Supplementary figures and images for: Epilepsy in Dcx Knockout Mice Associated with Discrete Lamination Defects and Enhanced Excitability in the Hippocampus
Source: PLoS One. 2008 Jun 25;3(6):e2473. doi: 10.1371/journal.pone.0002473 (PMC2429962; doi:10.1371/journal.pone.0002473)

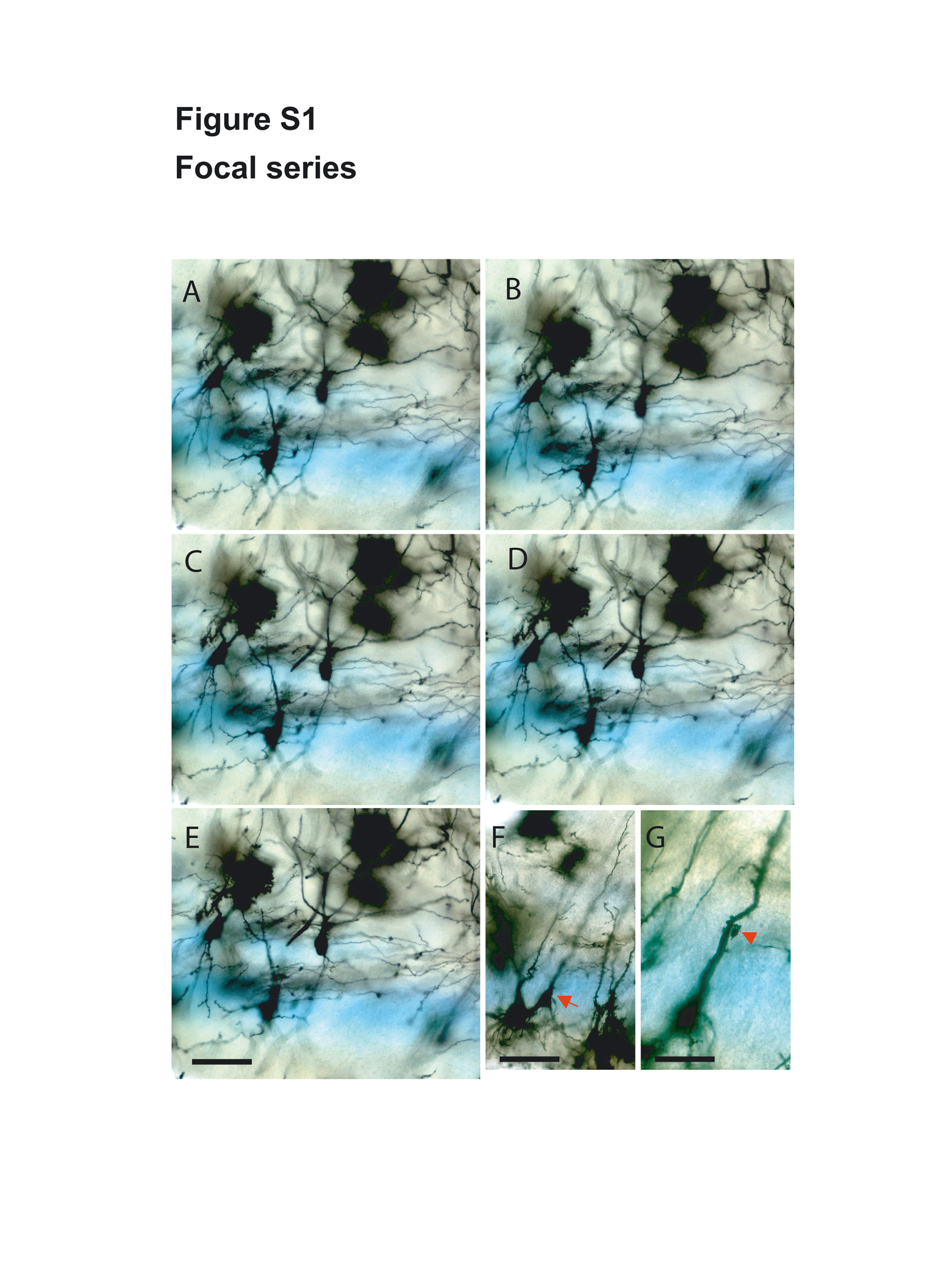

Supplement: Figure S1 — A focal z series of interneuron-like cells shown in Fig 1H, compared to a CA3 pyramidal cell in the same hippocampal section. (A–E) The Golgi-Cox stainings were performed in 40 µm brain slices and the three soma of interneuron-like cells are not all in focus in the same plane. This focal series helps show that each cell shows the typical morphology of a CA3 basket cell or axo-axonic-like interneuron [14], with fusiform cell bodies located within or adjacent to the pyramidal cell layer, and one or two dendrites originating from the apical pole, which then branch proximally to give radially oriented dendrites in the stratum radiatum. In addition, such cells have several basal dendrites branched close to the cell body and extended toward the alveus. Spines are rarely present on these few branches. Despite this recognisable morphology, we cannot specifically say what type of interneurons these are, because of the absence of a labelled axon arbor. (F, G) CA3 pyramidal cells (arrow in F and same cell shown in G) differ from this because they have one prominent apical dendrite emerging from a triangular soma, and this is radially oriented in the stratum radiatum where it is branched into large diameter segments, and in the stratum lacunosum moleculare where they emit several branches. Basal dendrites are also numerous in the stratum oriens. In addition the dendritic tree is typically densely covered with spines. The proximal apical dendrite has large complex spines (the thorny excrescence, arrowhead G) which form complexes with the large mossy fiber terminal. Scale bar E (for A-E); F, 75 µm; G, 37.5 µm. (17.83 MB TIF) [file pone.0002473.s004.tif]

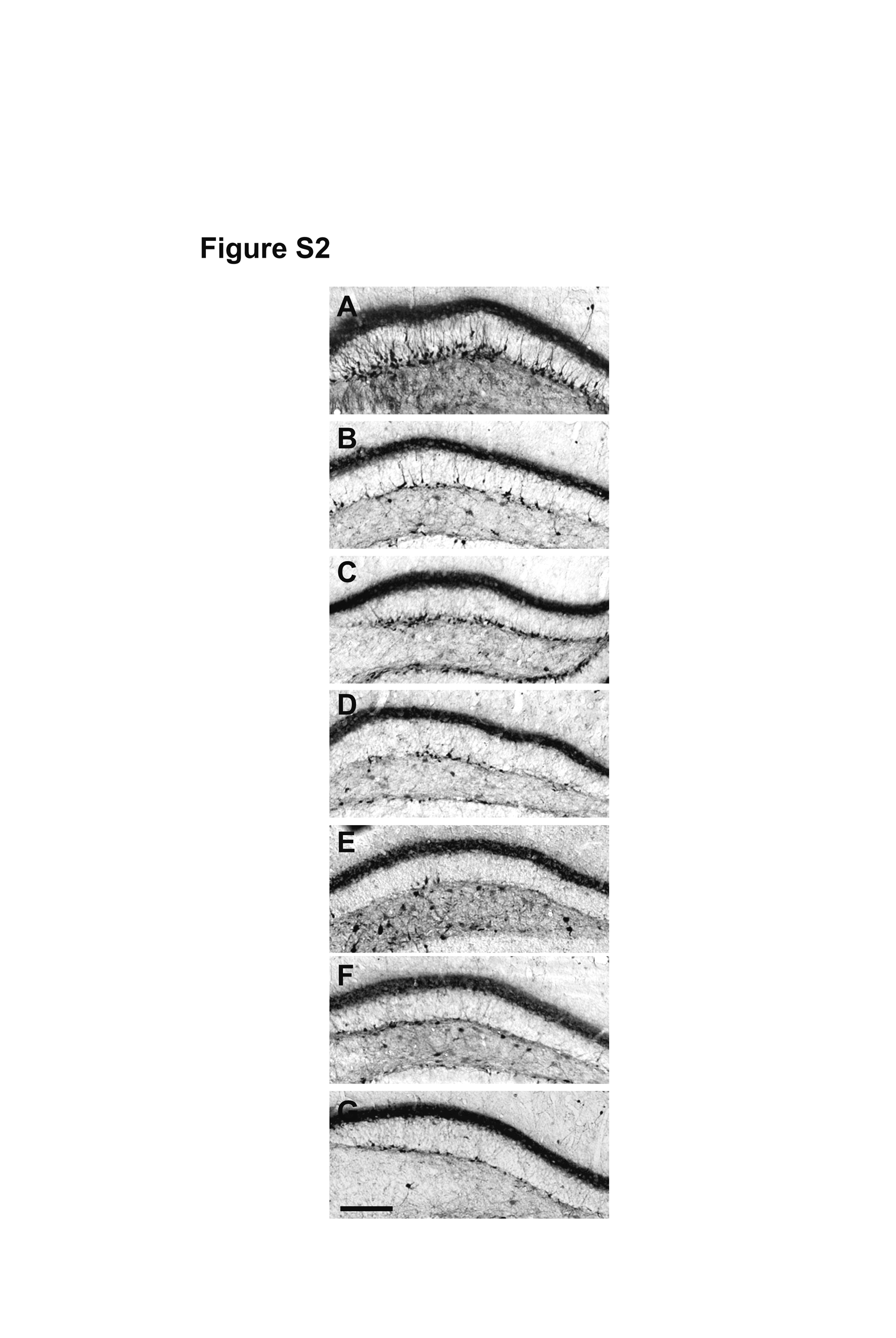

Supplement: Figure S2 — Changes observed in calretinin -positive cells in the subgranular zone of the dentate gyrus in Dcx KO animals. (A) Two KO animals were observed to have a greatly augmented number of disorganized calretinin-positive cells, as shown here for one animal. Both animals also showed changes in NPY and CB. (B, C) Two further KO animals showed increased and/or disorganized calretinin-positive cells. The animal in B also showed changed NPY and CB. (D, E) Certain KO animals (n = 3) showed subtly disorganized calretinin-positive cells which were however not visibly increased number. The animal shown in D did not show changes in NPY and CB, whereas two further animals (one of which is shown in E) did. (F) Two KO animals, one of which is shown here, showed no overt changes in calretinin-positive cells, and no changes in NPY and CB. (G) A WT section is shown for comparison. 4 WT animals were analyzed. Scale bar G (for A–G), 100 µm. (5.83 MB TIF) [file pone.0002473.s005.tif]
